# Impact of surgeons’ experience on accuracy of radiographic segmental kyphosis assessment in thoracolumbar fractures: a prospective observational study

**DOI:** 10.1186/1754-9493-8-15

**Published:** 2014-03-17

**Authors:** José Alexandre Lopes da Silva Alvarenga, Delio Eulalio Martins, Renato Hiroshi Salvioni Ueta, David del Curto, Marcelo Wajchenberg, Eduardo Barros Puertas

**Affiliations:** 1 Department of Orthopedics and Traumatology, Escola Paulista de Medicina-Universidade Federal de São Paulo (Unifesp), São Paulo, SP, Brazil

**Keywords:** Kyphosis, Radiography, Thoracolumbar fracture

## Abstract

**Background:**

The thoracolumbar region is where most fractures of the spine are located. Segmental kyphosis is an important factor for treatment decisions. There are various methods for measuring segmental kyphosis in thoracolumbar fractures. Our objective was to evaluate if the experience of the surgeon has any influence on kyphosis measurement by analyzing three different categories of orthopedic surgeons and evaluate possible clinical impacts.

**Material and methods:**

Six physicians separated into three categories according to the level of experience evaluated 30 lateral view radiographs of the thoracic spine of patients with single-level fracture taken during their outpatient follow-up visits. Images had segmental kyphosis measured by five distinct methods. The x-rays were evaluated twice and in a random order after an eight-week interval. The reproducibility of the measurements was analyzed by the intraclass correlation coefficient (ICC) and its respective 95% confidence interval.

**Results:**

The intraclass correlation coefficient (ICC) was calculated to evaluate the inter- and intra-examiner reliability for each method. The methods that disregard the fractured vertebra (1 and 4) achieved the highest intra and inter-observers reliability among the participants. The measurements from methods 3 and 5 were poorly reproducible between examiners. The difference between the averages of the measurements of the five methods studied was greater than 5 degrees in methods 1 and 2, suggesting risk for patient safety.

**Conclusion:**

Methods that exclude the fractured vertebra were more reproducible for the evaluation of segmental kyphosis in thoracolumbar fractures. The evaluation of the spine fracture must be coupled with other radiographic criteria, more complex image exams and the patient’s clinical state to assist the surgeon in deciding between conservative or surgical treatment. The authors suggest that the measurements should be performed by methods that exclude the fractured vertebra and conducted by experienced doctors.

## Background

The thoracolumbar transition is a region of biomechanical stress and is the location of most of the fractures of the spine [1]. The predominance of fractures at this location is explained by the contrast between the rigid thoracic kyphosis and the flexible lumbar lordosis. These fractures are bimodal and occur predominantly in young males after high energy trauma and the elderly after minor trauma. The main causes of these fractures are vehicle accidents, falls and sports trauma. A high incidence due to gunshot wounds has also been observed [2,3].

Several radiographic parameters are used to guide treatment and to determine the prognosis of these lesions, such as sagittal alignment of the affected segment, percentage of spinal canal compromise, translation of the vertebral body and scoliosis of the region involved.

There are various methods for measuring segmental kyphosis after thoracolumbar fractures, such as Cobb angle measurement, Gardner’s method, wedging of the fractured vertebra and others, but none of these methods has been evaluated for reproducibility between groups of surgeons with different levels of experience. These measurements have extreme importance in the initial evaluation of patients, and the therapeutic decision is strictly linked to reliable and reproducible measures.

Studies report that a segmental kyphosis greater than 30 degrees is most likely a consequence of posterior ligamentous complex disruption, which indicates surgical treatment of the fracture [4-6]. However, the angle value may differ depending on the technique used or the experience of the surgeon. The present study aimed to evaluate and compare five measurement methods of segmental kyphosis obtained by different experience categories of orthopedic surgeons to determine if the level of experience has an impact on these measures and if the fracture morphology affects the measurement methods.

## Methods

This study was previously submitted and approved by the ethical committee of this institution.

A sample calculation was performed using the intraclass correlation coefficient and SPSS® version 17.0. Thirty lateral view radiographs with single fractures between T11 and L2 were evaluated.

All 30 patients included in the study were seen during outpatient follow-up visits at this institution. The professionals who participated in this study considered the printed images of good quality, and there was no need to carry out additional tests, as the images were used as part of the initial assessment of the trauma patient.

The images were evaluated by different classes of professionals in the field of Orthopedics and Spine Surgery, including two members in each of the following categories: orthopedics residents with up to three years of experience, named OR; specializing fellows in spinal surgery with up to five years of experience, named SF; spinal surgeons with at least ten years of experience, named SS.

**Figure 1:**
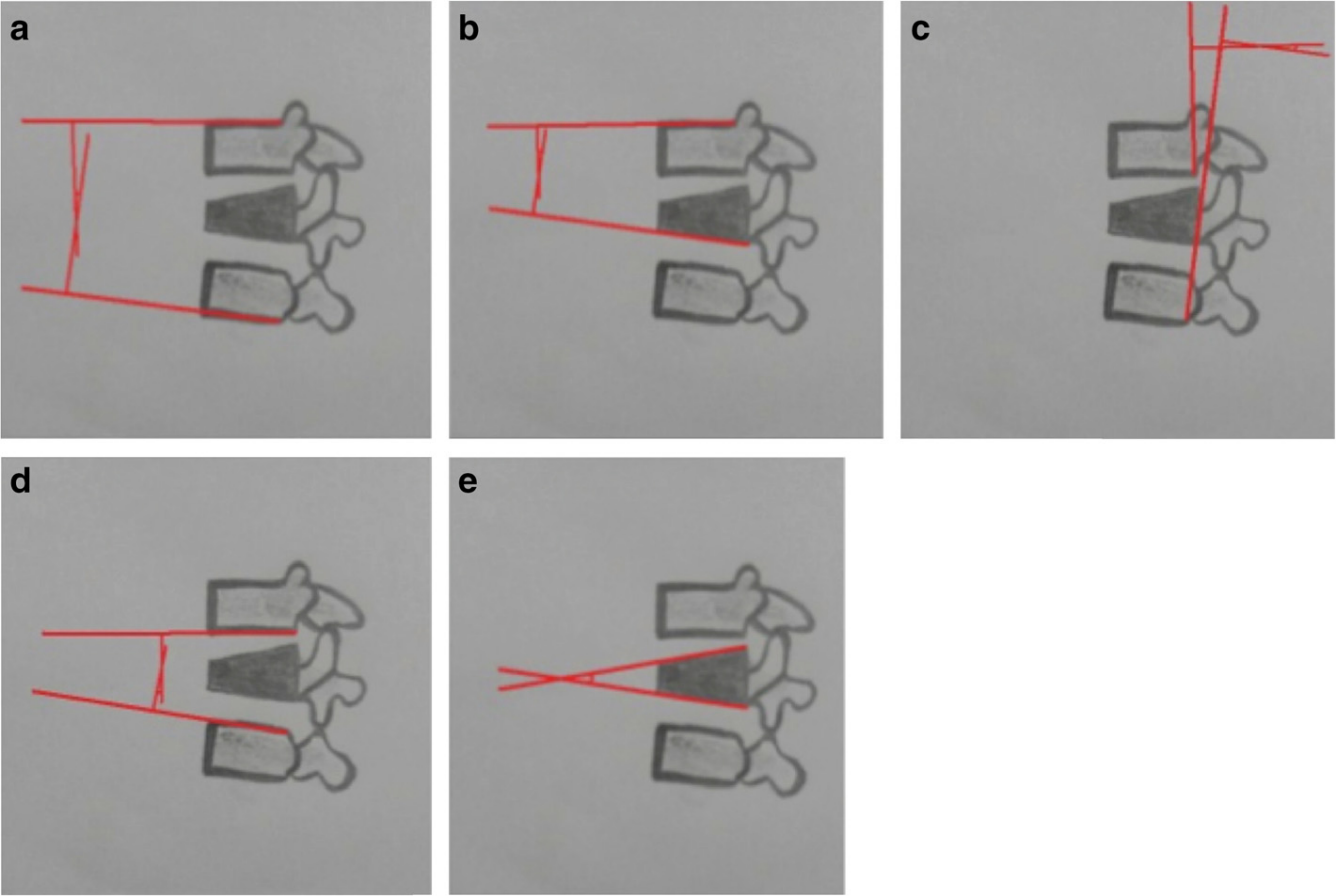
**Five methods described in the literature for kyphosis measurement on thoracolumbar fractures. a)** method 1, **b)** method 2, **c)** method 3, **d)** method 4 and **e)** method 5.

For measuring kyphosis, five methods described in the literature were used [7-12]: the first method (Figure 1a) uses the superior endplate of the vertebra above and the inferior endplate of the vertebra below the fractured one, yielding the Cobb angle. The second method (Figure 1b) uses the upper endplate of the vertebra above and the lower endplate of the fractured vertebra, yielding the Gardner segment deformity; the third method (Figure 1c) uses the angle measured between the posterior walls of the vertebral bodies above and below the fracture; the fourth method (Figure 1d) measures the angle between the lower plateau of the vertebra above and the upper plateau of the vertebra below the fracture; and the fifth method (Figure 1e) measures the angle formed from the upper and lower plateaus of the fractured vertebra.

Examiners received a brief introductory training prior to the first rating session. Each examiner received printed radiographs numbered from one to thirty and a sheet containing a draft of the five techniques to evaluate segmental kyphosis (Figure 1), in addition to pencils, an eraser and a standard goniometer.

After the first measurement, the x-rays were randomly set in a different order, and a new measurement of the participants was requested after eight weeks.

Patients with pathological fractures and those who had more than one level spine fracture were excluded.

## Results

The intraclass correlation coefficient (ICC) and its respective 95% confidence interval were calculated to evaluate the inter- and intra-examiner reliability for each method.

### Intraclass correlation

OR 1 had the greatest correlations with methods 1 and 2 and the smallest angular differences between the first and second measurements with methods 1 and 4. OR 2 obtained the greatest correlations and the smallest angular differences with methods 1 and 4. The third method yielded the lowest correlation and the greatest angular difference between the two measurements (Table 1).

**Table 1:** Intra-examiner agreement among resident physicians

**Examiner**	**Method**	**Measurement**	**Mean**	**SD**	**ICC**	**CI (95%)**	**Absolute difference**	
							**Mean**	**SD**
OR 1	1	First	12.23	7.45	0.965	0.929 - 0.983	1.47	1.14
		Second	11.90	6.47				
	2	First	16.00	8.67	0.946	0.875 - 0.976	2.13	1.91
		Second	14.80	8.56				
	3	First	9.43	7.25	0.768	0.490 - 0.893	4.00	2.41
		Second	7.10	5.96				
	4	First	8.27	7.40	0.942	0.882 - 0.972	1.73	1.60
		Second	7.60	6.31				
	5	First	13.83	8.71	0.934	0.818 - 0.972	2.80	1.45
		Second	12.23	8.43				
OR 2	1	First	11.40	6.31	0.937	0.874 - 0.970	2.03	0.96
		Second	11.77	6.37				
	2	First	15.63	8.12	0.882	0.769 - 0.942	3.27	2.05
		Second	16.57	7.67				
	3	First	12.67	5.57	0.415	0.062 - 0.673	5.30	3.03
		Second	12.57	5.78				
	4	First	9.43	6.06	0.958	0.915 - 0.980	1.50	0.94
		Second	9.33	6.17				
	5	First	13.80	7.30	0.776	0.584 - 0.886	3.90	2.80
		Second	14.97	6.90				

*OR*, Orthopedics resident.

SF 1 yielded the highest correlations and the smallest angular differences between the first and second measurements with methods 1 and 2. SF 2 showed higher concordance in methods 1 and 2 as well, but the smallest angular differences between the two measurements were obtained with methods 1 and 4. Method 3 also had the lowest intraclass reproducibility (Table 2).

**Table 2:** Intra-examiner agreement among spine surgery fellows

**Examiner**	**Method**	**Measurement**	**Mean**	**SD**	**ICC**	**CI (95%)**	**Absolute difference**	
							**Mean**	**SD**
SF 1	1	First	12.43	6.22	0.970	0.930 - 0.986	1.37	0.72
		Second	11.80	6.28				
	2	First	17.73	9.94	0.938	0.873 - 0.970	2.77	1.96
		Second	16.77	9.17				
	3	First	10.43	6.97	0.843	0.698 - 0.922	3.60	2.06
		Second	9.83	7.77				
	4	First	9.40	6.75	0.824	0.665 - 0.912	2.83	2.57
		Second	8.83	6.06				
	5	First	14.50	10.08	0.912	0.823 - 0.957	3.33	2.28
		Second	14.37	9.04				
SF 2	1	First	11.60	6.21	0.951	0.900 - 0.976	1.67	0.80
		Second	11.27	5.50				
	2	First	15.33	9.15	0.891	0.781 - 0.947	3.57	2.05
		Second	14.03	8.31				
	3	First	12.53	5.81	0.517	0.174 - 0.742	4.73	2.80
		Second	9.87	4.70				
	4	First	9.27	5.61	0.863	0.732 - 0.932	1.80	1.88
		Second	9.20	4.18				
	5	First	14.30	8.45	0.804	0.628 - 0.901	4.10	2.68
		Second	13.80	7.10				

*SF*, Specializing fellows.

The SS showed more uniform results, and the greatest correlations were observed for methods 1 and 4. The smallest angular differences between the first and second measurements were also obtained with these methods. Once again, method 3 had low reproducibility and the largest angular difference (Table 3).

**Table 3:** Intra-examiner agreement among spinal surgeons

**Examiner**	**Method**	**Measurement**	**Mean**	**SD**	**ICC**	**CI (95%)**	**Absolute difference**	
							**Mean**	**SD**
SS 1	1	First	11.77	6.42	0.946	0.890 - 0.974	1.93	0.91
		Second	12.37	6.56				
	2	First	15.40	8.18	0.903	0.805 - 0.953	3.43	1.36
		Second	16.50	8.51				
	3	First	8.03	6.01	0.594	0.302 - 0.784	4.20	3.36
		Second	8.70	5.88				
	4	First	8.47	5.62	0.966	0.922 - 0.985	1.40	0.50
		Second	9.07	5.75				
	5	First	14.10	7.27	0.837	0.686 - 0.919	3.40	2.28
		Second	13.70	7.03				
SS 2	1	First	12.77	7.02	0.978	0.950 - 0.990	1.17	0.83
		Second	12.20	6.69				
	2	First	17.00	9.46	0.916	0.801 - 0.962	3.30	1.56
		Second	15.37	8.05				
	3	First	10.23	6.88	0.802	0.627 - 0.900	3.60	1.79
		Second	9.17	5.76				
	4	First	9.40	6.48	0.982	0.961 - 0.991	0.97	0.72
		Second	9.03	6.03				
	5	First	14.73	7.86	0.867	0.687 - 0.940	3.33	1.77
		Second	12.93	6.46				

*SS*, Spinal surgeons.

### Interclass correlation

Methods 1 and 4 showed greater reliability among SF and SS, being superior to others in these two groups of examiners.

As for the OR group, method 2 demonstrated the greatest interclass reliability, followed by method 1. Method 3 had the lowest correlation and was inferior to the other methods in all categories of evaluation in this study (Table 4).

**Table 4:** Inter-examiner correlation

**Examiner**	**Method**	**ICC**	**CI (95%)**	**Absolute difference**	
				**Mean**	**SD**
OR	1	0.779	0.589 - 0.888	3.57	2.93
	2	0.885	0.772 - 0.943	3.23	2.45
	3	0.478	0.143 - 0.714	5.77	3.96
	4	0.683	0.438 - 0.835	4.43	3.13
	5	0.600	0.308 - 0.788	5.50	4.62
SF	1	0.867	0.741 - 0.934	2.50	2.05
	2	0.764	0.552 - 0.882	5.00	4.44
	3	0.636	0.358 - 0.809	4.23	3.71
	4	0.857	0.720 - 0.929	2.60	2.09
	5	0.702	0.459 - 0.846	4.33	5.78
SS	1	0.938	0.858 - 0.972	2.00	1.31
	2	0.894	0.774 - 0.950	3.47	2.22
	3	0.626	0.342 - 0.804	4.13	4.02
	4	0.944	0.861 - 0.975	1.53	1.38
	5	0.866	0.740 - 0.934	3.30	2.14

*OR*, Orthopedics residents; *SF*, Specializing fellows; *SS*, Spinal surgeons.

**Figure 2:**
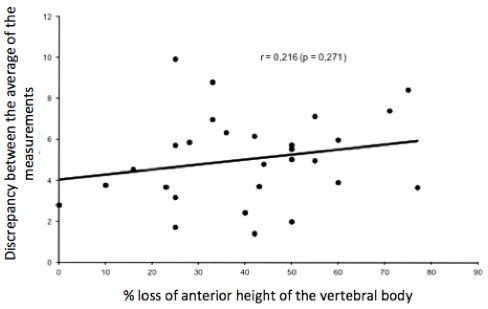
Percentage of loss of vertebral body height and the angular variation carried out by Spine Surgeons (p = 0.271).

The correlation value obtained between the variability of the averages of the methods by the experts (standard deviation between the methods) and the percentage of height loss was r = 0.216 (p = 0.271). Therefore, there is no statistically significant relationship between the loss of vertebral body height and the discrepancy between the averages of the measurements of each method (Figure 2).

The difference between the averages of the measurements of the five methods studied was calculated (Table 5). Among some methods, this difference was greater than 5 degrees, suggesting risk to patient safety because there could be surgical indication if the kyphosis was considered alone. However remember that other criteria are critical for surgical indication.

**Table 5:** Diference among measures

	**Diference among measures**														
	**Method 1**			**Method 2**			**Method 3**			**Method 4**			**Method 5**		
	**SS - OR**	**SS - SF**	**OR - SF**	**SS - OR**	**SS - SF**	**OR - SF**	**SS - OR**	**SS - SF**	**OR - SF**	**SS - OR**	**SS - SF**	**OR - SF**	**SS - OR**	**SS - SF**	**OR - SF**
Mean	5,16	5,93	0,80	0,45	7,74	0,08	0,06	-0,53	-0,57	0,51	1,02	0,53	-0,61	-1,18	-0,50
SD	2,56	5,93	2,34	1,40	3,30	1,34	1,17	1,81	1,33	0,74	0,68	0,32	1,28	1,10	1,27

*OR*, Orthopedics residents; *SF*, Specializing fellows; *SS*, Spinal surgeons.

## Discussion

In this study, methods 1 and 4 were more reproducible among most of the participant surgeons. We believe that the comparison of measurement methods between examiners with different levels of experience adds a key differentiator to this study because in most University services, the first evaluation is performed by professionals who are not specialists in spine surgery.

Kuklo *et al*. [8] compared different methods of measurement of segmental kyphosis in thoracolumbar fractures but did not carry out comparative analysis between the measurements performed by examiners with different levels of experience. They compared the measurements performed by two orthopedists and a neurosurgeon, noting that the Cobb angle method was more reliable within and between groups of examiners.

Plain radiograph is the first imaging modality used in trauma and should provide important information that, when combined with other tests of greater complexity and with clinical examination of the patient, should indicate the most appropriate therapeutic protocol. Post-traumatic kyphosis is an important indicator of prognosis and treatment of thoracolumbar fractures because an increase in this angle is directly related to the instability of the fracture [13-15]. In addition, studies report a possible association between the kyphotic deformity and residual back pain, making this a crucial element in the indication of surgical treatment for these fractures [16-18].

We observed that the methods which disregard the fractured vertebra (methods 1 and 4), were more uniform and consequently had greater agreement within and between groups of professionals. This is because some fractures involve one of the vertebral endplates or cause large body destruction, making it difficult to determine the correct adjacent lines, leading to results with high angular variability. The methods that take into consideration the fractured vertebra are prone to mistakes and variability, but in most thoracolumbar fractures, the upper plateau is more affected than the lower plate. Therefore, the second method was found to have good reliability. The measurements from methods 3 and 5 were poorly reproducible between examiners.

**Figure 3:**
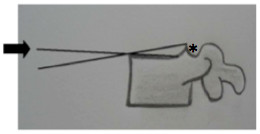
We propose drawing the line parallel to the flat surface of the body in such cases (black arrow) and ignoring the upper endplate ridge (asterisk).

Another factor that can lead to errors during measurement is the presence of osteophytosis in the posterior region of the vertebral plateau. The measurement may change due to the presence of a bony prominence that often distorts the flat surface of the endplate causing crucial errors [10]. This was one of the factors causing the most disagreement among the residents. Therefore, it is recommended to ignore this posterior bone elevation often found in x-rays (Figure 3).

**Figure 4:**
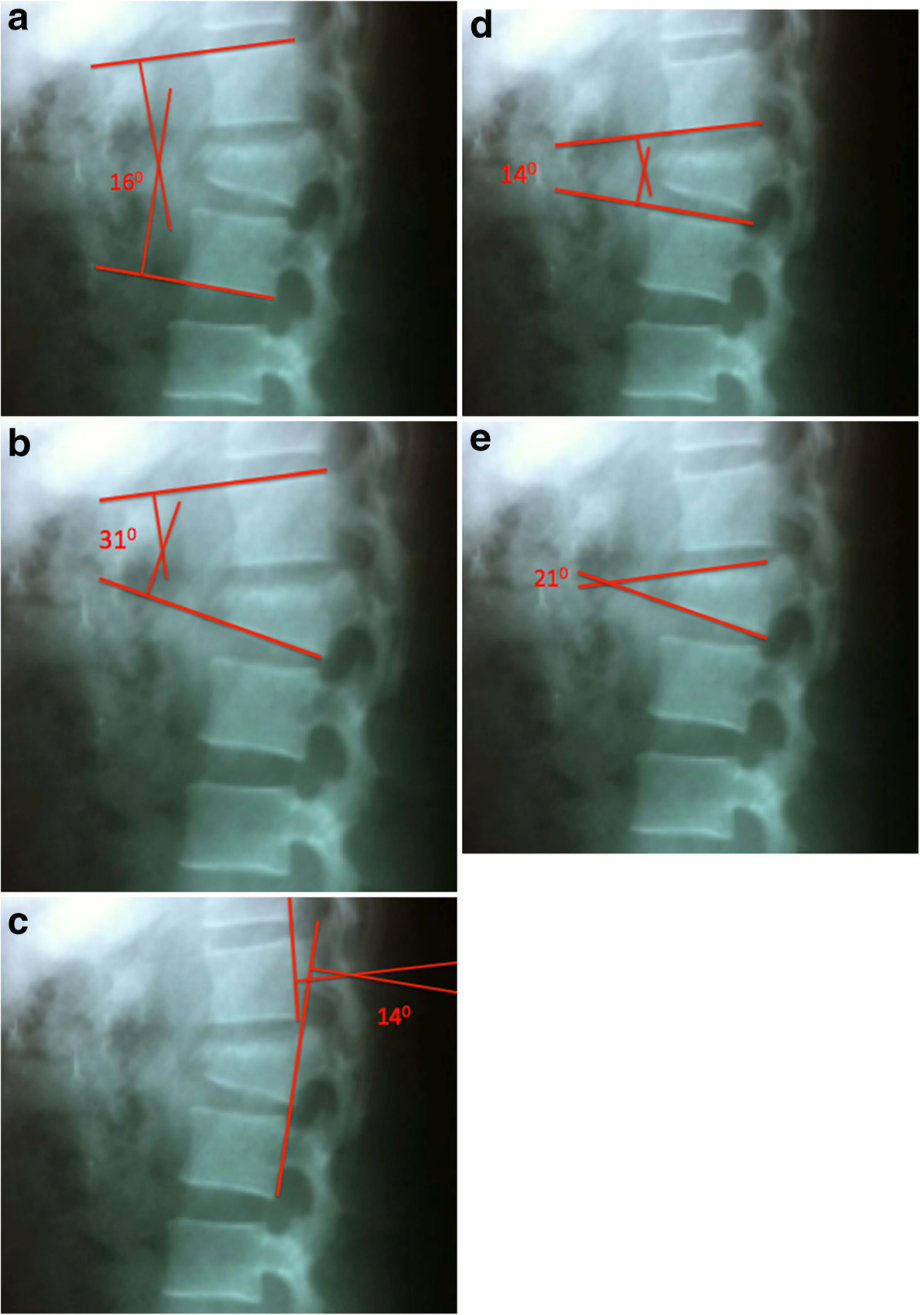
**Radiography of the thoracolumbar transition with L1 fracture.** Evaluation of segmental kyphosis by the five methods described: **a)** method 1, **b)** method 2, **c)** method 3, **d)** method 4 and **e)** method 5.

An example used in this study shows L1 fracture in a patient victim of an automobile accident. The segmental kyphosis was measured by the five methods described previously. Figure 4 shows the results of measurements performed by a spine surgeon. In this case, only method 2 (Figure 4b) showed segmental kyphosis greater than thirty degrees and all other methods showed lower values of kyphosis (Figure 4a,c,d and e). Since the patient had no other signs of instability, and the most reliable methods (1 and 4) showed regional kyphosis lower than thirty degrees, conservative treatment was prescribed. The patient was treated with Jewett vest for 12 weeks with fracture union and no complications.

Currently, the TLICS score (Thoracolumbar plate Injury Classification and Severity Score) proposed by Vaccaro *et al.*[19] provides a new perspective in the evaluation of fractures and helps with the therapeutic decision. It is based on the morphology of the fracture, ligament and disk complex injury and the neurological status of the patient. Radiographic analysis provides important data that suggest the severity of the injury and indirectly provide its prognosis, but it must always be complemented by other image tests, a detailed history that includes the trauma mechanism, a neurological examination and the patient’s comorbidities to ultimately determine the best form of treatment.

Therefore, we believe that the evaluation of segmental kyphosis from lateral x-rays of the spine must be coupled with other radiographic criteria, more complex image exams and the patient’s clinical state to assist the surgeon in deciding between conservative and surgical treatment.

## Conclusion

Methods 1 and 4 were more easily reproducible for the evaluation of segmental kyphosis in thoracolumbar fractures among the examiners who participated in the study. No relationship between the loss of anterior vertebral body height and the discrepancy between the averages of the measurements of each method was observed.

The improvement of measurement methods makes it possible to obtain more reliable measures, regardless of the surgeon’s experience, facilitating communication and homogenizing decisions. The authors suggest that the measurements should be performed by methods that exclude the fractured vertebra and conducted by experienced doctors.

## Consent

Written informed consent was obtained from the patient for the publication of this report and any accompanying images.

## Competing interests

The authors declare that there are no conflicts of interest including financial, consultant, institutional and other relationships.

## Authors’ contributions

The work presented here involved the collaboration of all authors. JALSA, DEM and MW defined the research theme and designed the methods, collected and analyzed the data, interpreted the results and wrote the paper. RHSU, DC and DEM worked on interpretation and discussed the analyses, interpretation, and presentation. EBP gave critical and final approval. All authors have contributed to, seen and approved the manuscript.
